# Mucosal Leishmaniasis in Travelers with *Leishmania braziliensis* Complex Returning to Israel 

**DOI:** 10.3201/eid2504.180239

**Published:** 2019-04

**Authors:** Michal Solomon, Nadav Sahar, Felix Pavlotzky, Aviv Barzilai, Charles L. Jaffe, Abedelmajeed Nasereddin, Eli Schwartz

**Affiliations:** Sheba Medical Center, Tel Aviv, Israel (M. Solomon, N. Sahar, F. Pavlotzky, A. Barzilai, E. Schwartz);; Hebrew University–Hadassah Medical School, Jerusalem, Israel (C.L. Jaffe, A. Nasereddin)

**Keywords:** leishmaniasis, *Leishmania braziliensis*, *Leishmania viannia braziliensis*, mucosal leishmaniasis, cutaneous leishmaniasis, travelers, Bolivia, Israel, parasites, ML, CL

## Abstract

Mucosal leishmaniasis (ML) is a complication of New World cutaneous leishmaniasis (CL) caused mainly by *Leishmania (Viannia) braziliensis*. This retrospective study investigated all cases of ML caused by *L. (V.) braziliensis* in a tertiary medical center in Israel, evaluating the risk factors, clinical presentations, diagnosis, treatment, and outcome of mucosal involvement in ML caused by *L. (V.) braziliensis* in travelers returning to Israel. During 1993–2015, a total of 145 New World CL cases were seen in travelers returning from Bolivia; among them, 17 (11.7%) developed ML. Nasopharyngeal symptoms developed 0–3 years (median 8 months) after exposure. The only significant risk factor for developing ML was the absence of previous systemic treatment. Among untreated patients, 41% developed ML, compared with only 3% of treated patients (p = 0.005). Systemic treatment for CL seems to be a protective factor against developing ML.

In the past 2 decades, travel to South and Central America has increased among young adults from Israel, causing potential exposure to tropical diseases, including leishmaniasis (*1*). New World cutaneous leishmaniasis (CL), endemic in some parts of the Americas, is caused by *Leishmania viannia* and *Leishmania mexicana* species complexes. Infection with *L. viannia* complex, particularly *Leishmania (Viannia) braziliensis*, results in CL that tends to be persistent and may be further complicated by mucosal leishmaniaisis (ML) (*2*). ML is probably caused by early hematogenous or lymphatic spread from cutaneous lesions through parasites that infect and replicate within macrophages of the nasooropharyngeal mucosa, setting up a destructive inflammatory process. The interval from onset (or clinical resolution) of CL to clinical manifestations of ML typically is several years but may range from <30 days to decades (*3*).

Persistent nasal congestion or stuffiness is the most commonly reported ML symptom (*4*,*5*); associated manifestations may include coryza, epistaxis, tissue/scab expulsion, pruritus, mass sensation, blockage/obstruction, and hyposmia (*4*,*6*–*9*). Persons with ML may have oral or pharyngeal lesions, bleeding, or pain; dysphagia/odynophagia; or dysphonia. Isolated laryngeal disease, without involvement of other mucosal sites, may occur but is relatively unusual (*7*,*9*). Abnormalities of the paranasal sinuses (e.g., those detected by computed tomography) have also been reported (*5*).

Systemic antileishmanial drugs are often used to treat CL caused by *L. viannia* complex, not only to promote healing of the primary lesion but also to reduce the risk of developing ML (*2*,*10*). Risk factors for development of ML are considered to be large or multiple cutaneous lesions, male sex, lesions above the waist, head and neck localization, and longstanding skin lesions for which adequate systemic treatment has not been administered (*11*).

In this study, we describe a cohort of 145 travelers from Israel returning from the Amazon Basin of Bolivia with New World CL. Within this group, 17 travelers developed ML. We compared these case-patients to patients with CL returning from this region without mucosal involvement, thus highlighting the clinical aspects and identifying potential risk factors for developing ML and noting appropriate treatment management.

## Patients and Methods

We conducted a multicenter survey of patients who received a diagnosis of New World CL during 1993–2005 at 8 medical centers in Israel. We collected additional data for 2006–2015 from cases referred to the Center of Geographic and Tropical Medicine or to the Dermatology Clinic at the Sheba Medical Center in Tel Aviv. All patients with New World CL diagnoses were evaluated retrospectively.

Suspected CL was confirmed when cutaneous lesions (ulcers, nodules, or papules) clinically compatible with leishmaniasis were noted and >1 of the following tests were positive: a smear or biopsy specimen showing *Leishmania* amastigotes within a dermal or mucosal infiltrate, positive PCR assay for *Leishmania (V.) braziliensis*, or positive promastigote cultures (*12*). Suspected cases of ML were confirmed when either nasal or oral symptoms of ML were noted together with oral or pharyngeal lesions. All the patients were examined by otorhinolaryngology specialists. Diagnoses were confirmed by biopsy, PCR, or culture for leishmaniasis.

Cure of a cutaneous lesion was defined as closure of the primary skin lesion. Cure of a mucosal lesion was defined as disappearance of the nasopharyngeal lesions.

### PCR Diagnosis

We performed DNA preparation and internal transcribed spacer 1 region (ITS1) PCR as described previously (*12*). In brief, we analyzed DNA samples at the time of original diagnosis for ITS1 PCR using primers LITSR and L5.8S. We performed the reaction with the PCR-Ready Supreme mix (Syntezza Bioscience, https://syntezza.com) in 25 μL of total reaction. Amplification conditions were as described previously (*12*). The PCR products were digested with *Hae*III enzyme for restriction fragment polymorphism analysis. The amplicons of ≈300–350 bp were analyzed on 1.5% agarose gels and the restriction fragments on 4% agarose gels by electrophoresis at 100 V in 1X Tris-acetate-EDTA buffer (0.04 M Tris-acetate and 1 mmol/L EDTA, pH 8) and visualized by UV light after being stained with ethidium bromide (0.3 μg/mL). We used GeneRuler DNA Ladder Mix (Thermo Scientific, https://www.thermofisher.com) as the DNA molecular marker. We also examined archived samples from 79 of the travelers in 2016 by HSP-70 PCR using the primers HSP70-F25 and HSP-70-R1310 (PCR-F) followed by DNA sequencing (*13*). We compared sequences to those in GenBank by using BLAST (https://blast.ncbi.nlm.nih.gov/Blast.cgi).

## Statistical Analysis

We used SPSS version 23.0 software (IBM, http://www.ibm.com/SPSS/Software) for data entry and analysis. Continuous variables were expressed as the median and interquartile range (IQR) and categorical variables as a percentage. We used a 2-tailed Fisher exact test to compute p value in the prevalence assessment. A p value <0.05 was considered significant.

## Results

**Figure 1:**
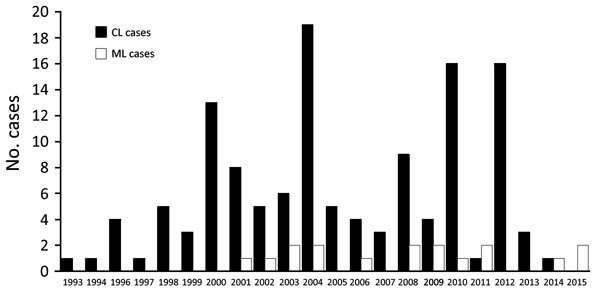
Number of CL and ML cases in Israel, 1993–2015. No cases were reported in 1995. CL, cutaneous leishmaniasis; ML, mucosal leishmaniasis.

During the past 22 years, 145 patients in our cohort received a diagnosis of CL from South America (Figure 1). From this cohort, 77 patients were seen during 1993–2005 in 8 medical centers in Israel (including Sheba Medical Center); the remaining 68 cases were a cohort of patients seen at Sheba Medical Center during 2006–2015. Of the cohort of 145 patients, 17 patients (16 men and 1 woman) received a diagnosis of ML (11.7%). All cases were acquired in known *L. (V.) braziliensis*–endemic areas in the Amazon region of Bolivia. 

**Figure 2:**
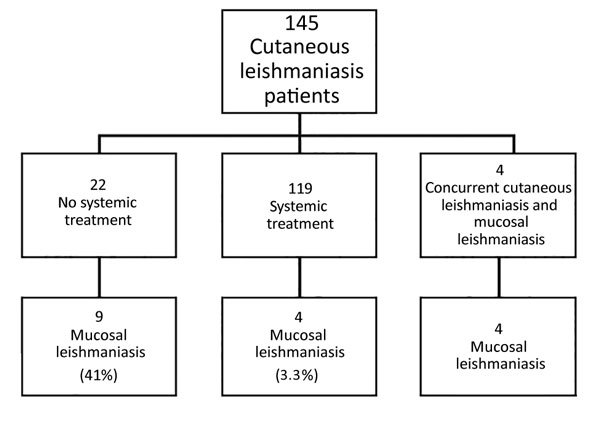
Outcomes of cutaneous leishmaniasis cases caused by *Leishmania (Viannia) braziliensis* based on treatment received, Israel, 1993–2015. In comparing the groups of patients, p = 0.005.

Of the 145 patients, 4 patients had concomitant cutaneous and mucosal lesions. Among the remaining 141 patients, 59 were treated with intravenous (IV) liposomal amphotericin B (L-AmB; 3 mg/kg/d for 6–10 d); 60 were treated with IV sodium stibogluconate (SSG; 20 mg/kg/d for 20 d); and 22 were not given systemic treatment for their primary skin lesion. Of those who were treated systemically, only 4 patients (3.3%) developed ML (3/60 among the IV SSG group and 1/59 among the IV L-AmB group), whereas in the group of 22 patients who were not given systemic treatment, 9 (41%) developed ML (p = 0.005) (Figure 2).

To explore other risk factors for developing ML, we compared patients with New World CL and those with ML (Table 1). The results showed no differences in age, sex, or number or location of skin lesions between the 2 groups.

**Table 1:** Comparison between patients with New World CL and those with ML, Israel, 1993–2015*

Characteristic	CL	ML
No. patients	128	17
Sex ratio, M:F	105:23 (82% male)	16:1 (94% male)
Mean age, y	24.2	27.6
Infected in Bolivia	83/100 (83%)	17/17(100%)
No. lesions	1.8	2.3
>3 lesions	21/128 (16%)	5/17 (29%)
Lesion above waist	61/81 (75%)	9/17 (53%)
PCR positive	68/76 (89%)	15/17 (88%)

*Differences between categories were not significant. CL, cutaneous leishmaniasis; ML, mucosal leishmaniasis.

We compiled epidemiologic characteristics and outcomes of the ML patients (Table 2). The mean age of the ML patients at diagnosis was 27.4 years (median 25 years, range 22–41 years). ML patients had a total of 42 skin lesions. The number of cutaneous lesions per patient was 2.47 (median 1, range 0–12); 53% had 1 cutaneous lesion, 29% had 2–4 cutaneous lesions, and 12% had >4 lesions. One patient had 12 lesions on his legs, which is rare for *L. (V.) braziliensis* infections, and 1 patient had no primary skin lesions. The distribution of the skin lesions was 29% on the upper limbs, 41% on the lower limbs, 23.5% on the face, and 11.7% on the trunk. Regional lymphadenopathy was found in 41% of the patients. All patients had negative serologic test results for HIV.

**Table 2:** Epidemiologic, clinical, and therapy data of patients with mucosal leishmaniasis, Israel, 1993–2015*

Patient no.	Age, y/ sex	No. primary lesions	Concurrent active CL	Location of primary lesions	Treatment				ML symptoms
					Primary cutaneous lesions	Mucosal lesions	After ML treatment failure	Response	
1	28/M	12	No	Trunk, upper extremities	None	IV SSG	No failure	CR	Oral ulceration, nasal obstruction
2	24/F	1	Yes	Lower extremities	Treated for concurrent CL	IV SSG	No failure	CR	Nasal obstruction
3	28/M	1	No	Lower extremities	None	IV L-AmB	No failure	CR	Nasal obstruction
4	28/M	1	No	Neck	IV SSG	IV L-AmB	No failure	CR	Nasal obstruction
5	26/M	1	No	Lower extremities	IV SSG	IV L-AmB	No failure	CR	Nasal obstruction
6	25/M	1	No	Face	IV SSG	IV L-AmB	No failure	CR	Oral ulceration
7	41/M	1	Yes	Lower extremities	Treated for concurrent CL	IV L-AmB	IV SSG	CL recurrence	Nasal obstruction, lacrimal gland obstruction
8	23/M	4	Yes	Neck, lower extremities	Treated for concurrent CL	IV L-AmB	No failure	None	Nasal obstruction, bone lesion
9	31/M	3	No	Upper and lower extremities	None	IV L-AmB	No failure	CR	Nasal obstruction, rhinorrhea
10	24/M	1	Yes	Upper extremities	Treated for concurrent CL	IV L-AmB	Miltefosine	CR	Nasal obstruction
11	41/M	0	No	No lesions†	None	Miltefosine	No failure	CR	Oral ulceration
12	25/M	1	NA	NA	None	IV SSG	No failure	CR	NA
13	22/M	2	No	NA	None	IV SSG	No failure	CR	Oral ulceration
14	25/M	3	No	Lower extremities	None	IV SSG	No failure	CR	NA
15	24/M	7	No	Face, upper extremities	None	IV SSG	No failure	CR	Oral ulceration, nasal obstruction
16	28/M	1	No	Upper extremities	None	IV L-AmB	No failure	CR	Nasal obstruction
17	23/M	2	No	Trunk	None	IV L-AmB	Miltefosine	CR	Nasal obstruction

*CL, cutaneous leishmaniasis; CR, complete response; IV L-AmB, intravenous liposomal amphotericin B; IV SSG, intravenous sodium stibogluconate; ML, mucosal leishmaniasis; NA, not available. †Patient 11 had no primary cutaneous lesion.

**Figure 3:**
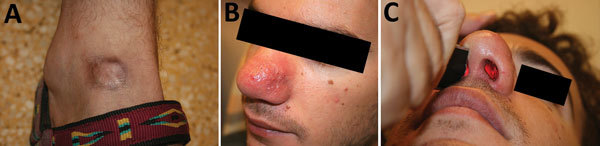
Cutaneous leishmaniasis and mucosal leishmaniasis in a traveler returning to Israel from Bolivia. A) Round hyperpigmented patch on the dorsum of right leg, representing old cutaneous leishmaniasis scar. B) Indurated erythematous patch of the nasal skin of the same patient appearing after 1 year. C) Illuminating in the right nostril sheds light into the left side, reflecting a hole within the nasal septum.

Nasopharyngeal symptoms developed 0–3 years after the patients returned from Bolivia (median 8 months), except for 1 case, which developed 20 years after exposure. ML diagnosis was delayed up to 5 years from the onset of symptoms (mean 16.3 months, range 0–60 months). Mucosal symptoms included oral ulceration in 5 patients, nasal obstruction in 12 patients, and lacrimal duct obstruction in 2 patients (in 1 patient, cartilage involvement of the sternoclavicular joint near the primary CL lesion was also noted). Typical nasal involvement is shown in Figure 3.

Regarding diagnosis, in 15 cases, the species diagnosis was based on positive PCR results for *L. braziliensis* taken by scraping of the lesion (mucosal or skin). In the other 2 cases, the species diagnosis could not be verified by PCR and was based on the disease being acquired in the Amazon basin of Bolivia, which is known to be endemic for *L. braziliensis*. Mucosal biopsy was done in 7 patients (41%) and revealed skin granulomas suggesting leishmanial infection, but amastigotes were seen in only 1 case, which demonstrates the limitation of biopsy in these cases.

Among the ML cases, 1 patient did not have a primary skin lesion, 4 patients had concurrent CL at time of diagnosis, and 12 patients developed mucosal symptoms after healing of the primary skin lesion. Among those 12 patients, symptoms developed 1 year (6 patients), 2 years (4 patients), or more (2 patients) after the initial diagnosis. Eight of those patients were not treated properly (they received paromomycin ointment, itraconazole, and/or intralesional sodium stibogluconate, or no treatment); 3 received IV SSG, and only 1 received IV L-AmB 3 mg/kg for 5 consecutive days and a 6th dose on day 10. The patient who did not have any skin lesions developed mucosal disease 20 years after returning from Bolivia.

### Treatment and Outcome

**Figure 4:**
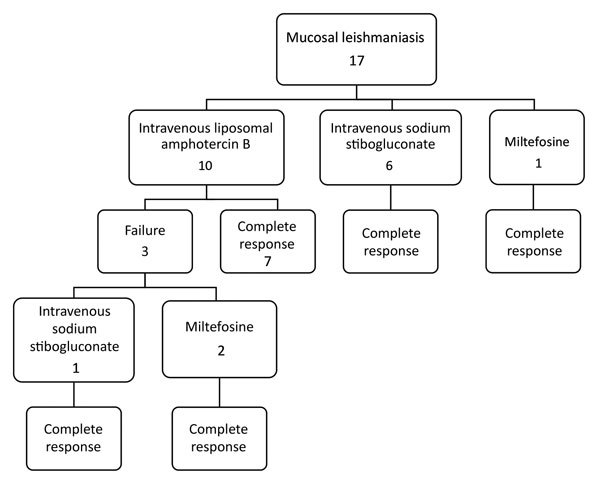
Treatment types and results for patients with mucosal leishmaniasis, Israel, 1993–2015.

Treatment of ML was carried out as follows: 10 patients received IV L-AmB at a dose of 3 mg/kg/day for 6–10 days (total 18–30 mg/kg); 6 patients received IV SSG at a dose of 20 mg/kg/day for 20–30 days; and 1 patient was given oral miltefosine at a dose of 150 mg/day for 28 days. With these doses, treatment failure with relapse occurred in 3 patients in the L-AmB group (Figure 4). Of the 3 patients whose ML failed to be cured with L-AmB therapy, 1 patient then received IV SSG and 2 patients were given oral miltefosine. The ML in all 3 of these patients was then cured. The rest of the patients achieved cures of their ML without having relapses, with a mean of follow-up of 9.5 years (median 7 years, range 2.5–16 years, IQR 5–11 years).

Two patients had irreversible nasal cartilage damage upon diagnosis at our center, with a hole in the nasal septum (Figure 3). These complications were a result of misdiagnosis and delayed proper treatment.

## Discussion

Our series describes 145 cases of CL in travelers returning to Israel; among these patients, 17 (11.7%) received diagnoses of ML. The highest-risk areas for ML are south of the Amazon basin in parts of Bolivia, Peru, and Brazil (defined here as the mucosal belt). Leishmania species with an increased risk of causing ML include mainly *L. (V.) braziliensis* but also *L. (V.) guyanensis* and *L. (V.) panamensis* (*3*). Among local populations, observational studies have generally found incidence rates of ML following CL caused by *L. (V.) braziliensis* to be 2%–10% (*14*) and close to 30% in some reports (*15*). In Bolivia, ML/CL ratios are highest (16%–37%) in the population living in endemic areas (*16*,*17*). Among indigenous persons in rural Bolivia with untreated CL, progression to ML was estimated to occur in 5%–20% of patients (*17*). Based on retrospective evaluations in an actively surveyed population of >3,000 CL patients in an *L. (V.) braziliensis* focus area in Peru, the lifetime risk of developing ML was 12.8% (*18*).

Few previous reports exist on ML among travelers returning from Latin America to non–*Leishmania*-endemic countries (*10*,*11*,*19*–*21*). An estimation of the ML/CL ratio in travelers with *L. (V). braziliensis* gives a range of 1.2%–8% (*14*). However, the prolonged follow-up period in our study provides a more firm basis for our finding of a rate of 11%.

Several *Leishmania* species are circulating in the Americas; therefore, the rate of ML might be different from region to region. Our study focused on *L. (V.) braziliensis* infection, notorious for causing ML complications. A report from a *Leishmania*-endemic area of Bolivia indicated that, compared with the indigenous population, healthy migrants to this region who developed CL had a 2.3-fold greater risk of developing ML (*22*). In this respect, travelers from non–*Leishmania*-endemic countries may similarly be more susceptible to ML. However, based on our data, it seems that the rate among travelers is similar to that of the local population.

The risk for ML following New World CL has been estimated to be highest within 2 years of the onset of the initial skin lesion (*9*). Indeed, 82% of the patients in our series developed ML symptoms within 2 years after onset of CL lesions.

Mucosal complaints in our study included nasal obstruction, rhinorrhea, nasal discharge, oral ulceration, bone lesion, and lacrimal duct obstruction. Lacrimal duct obstruction is less known; it is described in the literature in 4 patients, 20–75 years of age, who had nasal lesions resulting from ML and sought treatment for chronic dacryocystitis (*23*). However, based on our case series, invasion of the lacrimal ducts seems to be less uncommon (2/17, 11%).

Delay in diagnosis of ML was common in our study, which found a mean of 16 months from the onset of symptoms until appropriate treatment. A low index of suspicion by clinicians may have contributed to these delays. Increased medical awareness of the risk for CL and ML among travelers to Latin America may reduce delays in diagnosis and optimize chances of cure. Unfortunately, in 2 patients the disease was diagnosed too late, after the patients developed destructive mucosal lesions with a complete hole in the nasal septum that could not be cured (Figure 3, panel C). 

In our globalized world, ML should be considered in the differential diagnosis of granulomatous processes in biopsies taken from the nasopharynx, especially in returning travelers. Recent recommendations of the Infectious Diseases Society of America and the American Society of Tropical Medicine and Hygiene state that during all evaluations, persons at risk for ML should be questioned explicitly about the development, evolution, and other characteristics of mucosal symptoms (*3*). They should also undergo a thorough examination of the nasooropharyngeal mucosa by an otolaryngologist even if they do not have any mucosal symptoms. These patients should be educated about the importance of seeking medical attention for possible ML if they ever develop persistent, atypical nasooropharyngeal or laryngeal manifestations that do not have a clear etiology. The policy at Sheba Medical Center is to check all patients with cutaneous *L. (V.) braziliensis* for ML.

The factors that affect progression to ML are not clear but likely relate to the infecting *Leishmania* species; *L. (V.) braziliensis* is the species most strongly associated with ML (*3*). Other postulated risk factors for the development of ML include large lesions, multiple CL lesions, presence of lesions for >4 months, micronutrient deficiency, immunosuppression (*14*,*24*), location of lesions above the waist (*25*,*26*), and concentration of lesions on the head and neck. The explanation for the relationship between ML and CL lesions on the head and neck is that the proximity of the CL lesions to the head increases risk for developing ML because of the shorter distance that the parasite-laden macrophages must travel through lymphatic channels to reach the nasopharynx (*25*,*26*). However, in our series, none of these risk factors were found to be associated with progression to ML (Table 2). All our patients were young, healthy travelers with no evidence of impaired immunity and were negative for HIV.

One distinct risk factor is that CL patients without prior systemic treatment had a higher risk of developing ML (Figure 2). Of untreated patients, 41% developed ML, compared with 3.3% of treated patients. Thus, effective systemic treatment of New World CL caused by *Leishmania (Viannia)* species can decrease the risk for ML but may not prevent all cases of ML (*3*). As we mentioned, there was no failure of treatment of ML by IV SSG in our study, whereas 3 patients experienced failure of L-AmB treatment. However, this failure might be the result of a lower dosage; World Health Organization recently recommended 40–60 mg/kg of L-AmB, whereas our patients received the old regimen, which was about half this dose. We otherwise found no difference among the diverse regimens used for systemic treatment (i.e., IV L-AmB, IV SSG, oral miltefosine) in the outcome of the patients. Topical treatment is the most coemmon treatment for Old World cutaneous leishmaniasis. For New World CL caused by *L. braziliensis* complex, topical treatment recently has been discussed as a treatment option. However, our findings recommend using only systemic treatment in this infection because of the risk of development of ML (*27*).

This study has several limitations. First, the cohort included 2 parts. The first part was a multicenter study from 8 medical centers in Israel, the second patients referred to our tertiary center. As a result, we may have seen more complicated cases of New World CL, including cases of ML. In addition, most of the Israeli patients were returning travelers from Bolivia, which is endemic for *L. (V.) braziliensis*. Therefore, our findings may not represent all New World CL species.

In summary, our findings support that prolonged clinical follow-up of travelers returning from Bolivia with CL is likely warranted. CL in travelers from this region should be managed with systemic therapy according to clinical guidelines (*3*,*14*). Furthermore, we noted a high rate of ML in travelers with CL caused by *L. (V.) braziliensis*. Systemic treatment for CL seems to be a protective factor against developing ML. A high index of suspicion is required for prompt diagnosis of ML and optimal management to prevent irreversible damage.
